# Chironomid-climate continentality conundrum

**DOI:** 10.1371/journal.pone.0327780

**Published:** 2025-08-05

**Authors:** Varvara Bakumenko, Anneli Poska, H. John B. Birks, Brian Huser, Siim Veski

**Affiliations:** 1 Department of Geology, Tallinn University of Technology, Tallinn, Estonia; 2 Department of Physical Geography and Ecosystem Science, Lund University, Sweden; 3 Department of Biological Sciences and Bjerknes Centre for Climate Research, University of Bergen, Norway; Environmental Change Research Centre, University College London, London, United Kingdom; 4 Department of Aquatic Sciences and Assessment, Swedish University of Agricultural Sciences, Uppsala, Sweden; Centre National de la Recherche Scientifique, FRANCE

## Abstract

It is predicted that continentality, a climate parameter representative of a region’s annual temperature and precipitation range, will undergo significant changes in the future. The lack of past continentality reconstructions makes it impossible to decipher any long-term patterns of continentality changes. Here, we investigate the extent to which continentality influences modern chironomid assemblages and evaluate their ecological relevance for palaeolimnological data-based reconstructions of past continentality. We selected 53 lakes along a longitudinal gradient covering the East European Plain (Western part of Russia, Estonia, Latvia) and southern Scandinavia (Sweden and Norway). We analysed the dependency of chironomid assemblages on a variety of environmental parameters including two continentality indices (annual temperature range (ATR) and the Kerner Oceanity Index (KOI)), growing degree days at base temperature 5 °C, mean air temperatures of July, April, and October, number of ice-cover days, lake-water pH, loss-of-ignition and water depth using redundancy analysis. Correlations between all variables were tested to check for possible confounding effects. KOI had the highest explanatory power of 18.4% in the dataset and an absence of collinearity (correlation index < 0.7) with all the other tested variables. Further, we estimated weighted average optima to investigate the distribution of the morphotypes along the continentality gradient in the dataset. *Glyptotendipes pallens-*type, *Neozavrelia*, *Polypedilum sordens-*type, and *Microchironomus* showed a preference for a continental climate, while *Paratanytarsus penicillatus*-type, *Pseudorthocladius*, *Thienemannimyia*, and *Limnophyes* were found mainly in samples from oceanic areas. Weighted averaging-partial least squares regression was used for a trial test of the data, resulting in a promising KOI-based model performance with R^2^ = 0.73 and RMSEP = 5.1. Despite the relatively small dataset, our study suggests that chironomid data have the potential for further development as a tool for reconstructing palaeocontinentality.

## Introduction

Continentality, a climate parameter that combines information on annual variation in temperature and precipitation, has changed in the past and is predicted to change in the future [1,2]. Continentality of a region depends on the distance from the ocean and the prevailing atmospheric circulation patterns [2]. While the annual temperature range (ATR, calculated as the difference between the coldest and the warmest months) is the simplest and most used metric to estimate continentality, several other indices have been proven relevant to describe the continentality gradient in nature. These include the Gorzynski [3] continentality index, which includes latitude in the index, and the Kerner Oceanity Index (KOI; [4]), which includes October and April air temperatures.

Continentality has significantly changed over the last century [2,5], resulting in an increase in Northern Europe, most of North America and East Asia [2], and a decrease in the Eastern Baltic countries (Estonia, Latvia, Lithuania) [6]. These changes are expected to continue in the context of ongoing climate change [6,7]. Continentality variations may occur due to changes in solar radiation, and variations in atmospheric circulation and ocean heat transportation [8]. These changes affect various natural processes, such as permafrost degradation [9], ecosystem productivity [10], biodiversity distribution [11], tree bimodality growth [12]. Continentality can affect the aquatic zoobenthos by inducing variations in the start, duration, and heat accumulation of the growing season [13] as well as the timing of lake turnover [14]. It can also influence the formation and duration of the ice-cover at mid and high latitudes, leading to changes in water pH [15] and dissolved oxygen concentration [16]. Northern and eastern Europe, and the Baltic area in particular, are situated in a transitional zone between continental and oceanic climates, making this region highly suited for studies of long-term changes in continentality. Furthermore, a marked increase in continentality (annual temperature range) has been observed in the eastern Baltic area during recent decades (−1.7 KOI values per decade; [2]), highlighting the urgent need for past continentality-related knowledge, which could assist in making realistic, evidence-based continentality predictions. Understanding long-term continentality is essential for accurate climate modelling, ecosystem management, climate change adaptation, land-use planning, and unravelling the Earth’s geological history. It provides a framework for interpreting both past and future climate dynamics, especially in regions where the influence of landmasses dominates over oceanic moderation.

Long-term reconstructions of past changes in climate parameters are often used to determine and predict their impacts on ecosystems (e.g., [17–22]). Only a few reconstructions of palaeocontinentality have been published [23–27]. For instance, increased seasonality has been inferred from cryogenic cave carbonates in Great Britain during the Younger Dryas period event. [28], and from phosphorus concentrations changes in stalagmite calcite in western Ireland during the 8.2 ka cooling [29]. Other attempts to develop a continentality reconstruction have been based on tree rings or ice wedges data [25,27]. However, tree-ring data are confounded by too many intertwined environmental and climate factors, while ice wedges are limited to the few regions that historically had permafrost. Thus, not much is known about continentality changes in the past and their impact on ecosystems.

Larvae of chironomids, non-biting midges from the family Chironomidae, are recognised as one of the most reliable palaeoclimate proxies [30]. The taxonomic composition of subfossil chironomid assemblages is known to be responsive to environmental conditions [31,32], such as lake water trophic state [33,34], dissolved oxygen concentrations [35,36], pH [34,37], and depth [37,38]; warm season temperatures [39–42], and heat accumulation expressed as growing degree days (GDD; [43]). Chironomids are often assumed to be non-responsive directly to changes in winter temperatures as they experience diapause during the winter season in temperate and boreal climate zones [44]. However, several studies have shown a significant dependency of chironomid assemblages on winter temperatures [30,45]. An indirect impact of winter temperatures has been observed in several recent studies, showing that chironomid assemblages from boreal and temperate zones can be affected by the duration of ice cover, which is inversely correlated with dissolved oxygen levels and the warm season duration [24,46], as well as with continentality [47]. Self et al. [24] show that chironomid assemblages in northern Russia are influenced by continentality (Gorzynski continentality index), which is thought to have an indirect effect through variations in ice-cover period length. It is commonly recommended to use chironomid training sets only within the biogeographic area from which they originated [48,49]. Therefore, the training set developed by Self et al. [24] is applicable only in northern Russia.

**Fig 1:**
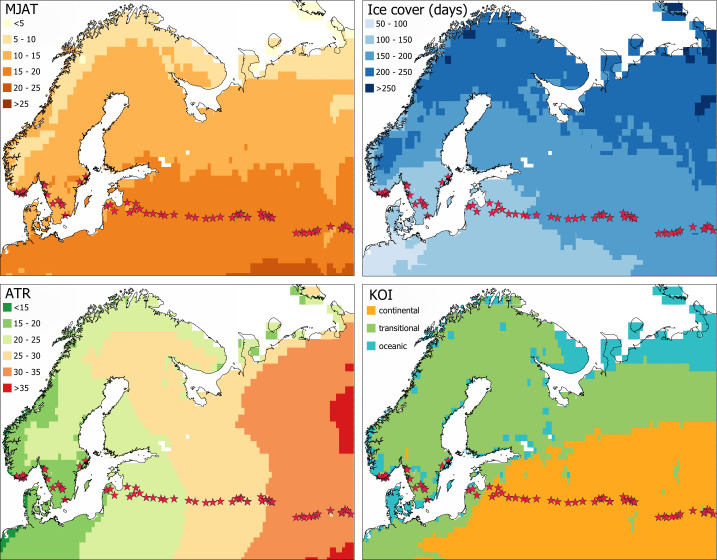
Map of sampled lakes with respect to mean July air temperature (MJAT; °C), ice-cover duration (days), annual temperature range (ATR; °C), and Kerner Oceanity Index (KOI; continental area (orange) is KOI −10 - 0, transitional (green) is KOI = 0–10, and oceanic (blue) is KOI 10-20).

To expand our understanding of the relationship between chironomids and continentality in areas with transitional climates in northern and eastern Europe, we have assembled a new chironomid dataset that represents a wide range of climatic and environmental variations along a longitudinal continentality gradient (Fig 1) in northern and eastern Europe – from the oceanic Norwegian coast to the continental Ural Mountains. The objectives of our study are to determine (1) possible confounding factors to continentality environmental variables; (2) the potential influence of continentality and related climate variables on chironomid assemblages; and (3) the indicator taxa representative of different parts of the continentality gradient. This paper aims to serve as a prerequisite and justification for the increased use of chironomids as a continentality proxy and as a starting point for developing a more extensive training set applicable in northern and eastern Europe. Data derived from continentality reconstructions will enhance our understanding of how continentality varies over time and how it impacts natural ecosystems.

## Materials and methods

### Climate data

Hourly temperature (^o^C) and lake ice-thickness (mm) data for each lake were extracted from the ERA5 dataset with hourly temporal and 0.25° x 0.25°spatial resolution [50], which was downloaded from the Copernicus Climate Data Store. Using the downloaded climatic data, the following variables were calculated based on a 30-year mean:

(1) Mean January, April, July and October temperatures (^o^C).
(2) Continentality indices: annual temperature range (ATR; the difference between the warmest month’s mean temperature (July for all sites) and the coldest month’s mean temperature (January for all sites)); Kerner Oceanity Index (KOI; [4]), reflecting not only annual temperature variation, but also the warmth of spring and autumn, calculated following the equation: KOI = 100×(To-Ta)/ATR, where To is the mean October air temperature (^o^C), Ta is the mean April air temperature (^o^C), and ATR is the annual air temperature range (^o^C)).
(3) Annual sum of Growing Degree Days at a base temperature of 5 °C (GDD5) was calculated by applying the daily temperature data following the equation [51]: $\sum_{i=1}^{365}\frac{Tmin+Tmax}{2}-Tbase$, where T_base_ equals 5 °C and i refers to day of the year.
(4) Ice conditions (number of ice-cover days): the number of ice-cover days was estimated using the ice-thickness dataset by summing the number of days with a minimum ice thickness across the lake > 0 mm.

Based on the above-listed datasets a set of thematic maps covering Northern and Eastern Europe was created. The dataset design strategy was guided by the longitudinal (east to west) continentality (ATR) gradient observed in northern Europe (Fig 1).

### Site selection

The following sets of sites (Table 1) were used to compile the training set:

**Table 1:** Environmental and climate gradients covered by the new combined dataset. Growing degree days and ice-cover represents sum annual variables.

Dataset origin	Norway	Sweden	Latvia	Russia
**Number of sites**	6	9	7	31
**Latitude (** ^**o**^ **N)**	58.02–58.25	57.2–59.5	56.4–57.3	54.5–56.2
**Longitude (** ^**o**^ **E)**	7.0–8.2	11.5–18.3	21.7–27.1	28.2–61.7
**Distance to sea (km)**	3.2–20.2	2–110	41.2–176	255–1568
**Elevation (m, above the sea level)**	24–251	28–238	51–108	57–391
**July air temperature (°C)**	14.6–16.8	16.0–17.3	18.0–20.0	17.8–20.2
**Annual temperature range (°C)**	14.4–16.7	17–19.4	19.8–22.7	23.0–33.3
**Kerner Oceanity Index**	15.8–27.5	6–11.3	−2.4–8.4	−4.5 – −1.6
**Growing degree days 5 (°C)**	1200–1511	939–1088	1599–1729	1497–1880
**Ice-cover period (days)**	35–83	43–65	91–126	126–196
**Sampling water depth (m)**	5.2–19.5	6.5–45	2.8–20	0.7–12.5
**Lake-water pH**	5.1–7	6–7.2	6.8–8.6	7.5–10.8

(1) six oceanic sites from Norway (part of the Swiss-Norwegian training set collected in 1995–1999 by Heiri et al. [39]; the data were downloaded from the National Centre of Environmental Information online storage);
(2) seven intermediate continentality sites from Latvia (part of the Finno-Baltic-Polish training set collected in 2019–2021 by Bakumenko et al. [42]);
(3) thirty-one intermediate to continental sites from western Russia collected for this study in 2021;
(4) nine oceanic to intermediate continentality sites from Sweden (Huser, unpublished) collected in 2014 were added to fill the biogeographical gap between the Norwegian and Latvian-Russian parts of the dataset (Fig 1).

Thus, the final dataset consists of 53 lake sediment surface samples along the longitudinal gradient 7–61.7 ^o^E, between 54.5 and 59.5 ^o^N latitude (Fig 1, Table 1). The dataset covers a broad range of environmental and climatic gradients. Most of the lakes are situated in low-elevation areas with fully vegetated catchments (Table 1). The surrounding biomes range from temperate steppe in the east to hemiboreal and temperate mixed in the central regions, and coniferous forests in the west. Bedrock includes sandstone in the eastern part of the dataset, limestone in the middle, and gneiss/granitoid in the western part (southern Norway and Sweden).

### Environmental data

The basic environmental variables (lake-water depth and pH, sediment loss-of-ignition (LOI), and catchment soil and bedrock composition) were included to assess their influence on the Chironomidae assemblages and to examine potential confounding effects with the climatic variables.

In connection with the surface-sediment sampling, lake-water depth and pH (at 30–40 cm above the sediment surface) were measured in the field. LOI was measured only for Russian and Latvian samples using the standard procedure [52].The underlying bedrock type was identified using a bedrock map of Europe (Commission of the Geological Map of the World Subcommission for Europe; CGMW). Soil composition data (sand, clay, and soil base saturation) were extracted from the FAO Digital Soil Map of the World (2003). While a multitude of other environmental parameters could affect the chironomid assemblages, we selected those that are most commonly used and consistently available across the entire dataset.

### Sediment sample collection and laboratory processing

Surface-sediment samples for all parts of the dataset were collected using a gravity corer from the deepest part of each lake. The upper 2 cm of lake sediment were taken for analysis. Sampling did not involve endangered or protected species and was done following legal acts of the corresponding countries. In the laboratory, sediment samples of 5 cm^3^ were water-sieved with a 100-µm mesh to remove fine sediment. Each sample was then transferred to a Petri dish from which chironomid head capsules were extracted with fine forceps under a stereomicroscope at 25x magnification. The head capsules were air-dried and mounted in Aquatex® or Euparal® mounting medium. Taxonomic identification was conducted under a light microscope at 100–400X magnification.

### Taxonomic identification and dataset harmonisation

For all four parts of the dataset (Norwegian, Swedish, Latvian, Russian), identification of the chironomid head capsules was done following the taxonomic approach of Brooks et al. [53]. Chironomid assemblages from the Latvian, Russian (analysed by Varvara Bakumenko), and Swedish (analysed by Simon Belle) parts were identified using keys by Klink and Pillot [54], Brooks et al. [53], Larocque-Tobler [55], and Andersen et al. [56]. On average, 69 chironomid head capsules were identified per sample, with a range of 47–139 (S1 File).Final taxonomic harmonisation was done after merging all the above-described datasets. All identifications at genus or subfamily taxonomic level (Tanytarsini, *Tanytarsus* spp., *Paratanytarsus* spp., Tanypodinae, Chironomini, Orthocladinae) were excluded from the merged dataset to avoid including broad groups of Chironomidae species with a wide range of ecological preferences. The excluded taxa made up less than 10% of the total head capsules count in the dataset and no more than 8% in any individual sample. *Cricotopus intersectus*-type was merged with *Cricotopus laricomalis*-type into one type due to the likelihood of misidentification of these morphotypes. Morphotype-level identifications from *Einfeldia (Einfeldia dissidens*-type), *Zalutschia (Zalutschia zalutschicola*-type), *Eukieferiella* (*Eukiefferiella coerulescens*-type), and *Dicrotendipes (Dicrotendipes nervosus*-type and *Dicrotendipes notatus*-type) were merged into corresponding genera level groups due to the differences in identification resolution in parts of the dataset. Harmonisation was done before transforming the data into relative abundances.

### Numerical analysis

The harmonized chironomid count data was transformed into relative abundances, and thereafter square-root transformed. To remove rare taxa, only morphotypes with an abundance higher than 2% in at least one sample were included for numerical analysis to improve performance of the inference model [57]. Based on KOI, the dataset was divided into 3 parts: continental with KOI −10–0, transitional with KOI 0–10, and oceanic with KOI 10–20 [2]. Analysis of similarities (ANOSIM; [58]) was applied to justify the KOI-based division of the chironomid assemblages. Bedrock data were grouped in 4 groups (sand-containing, clay-containing, carbonates-containing, granits/granitoids) and coded as numbers (1–4) for the numerical analyses.

Principal component analysis (PCA) was applied to the environmental and climate data of the dataset to investigate their gradients length. The Shapiro-Wilcox test and Spearman correlations were used to test for collinearity between environmental and climatic variables. Variables with correlation coefficient > ±0.7 were considered highly correlated and their effect on chironomid assemblages could not be distinguished from each other.

Detrended correspondence analysis (DCA; [59]) was applied to the chironomid assemblages data to examine the distribution of taxa and the compositional gradient lengths along the first two DCA axes. Redundancy analysis (RDA) was chosen based on the length of DCA axis-1 and axis-2 of the dataset (2.9 and 2 SD units, respectively; [60,61]). RDA was applied to determine which environmental variables explain significant compositional variation in the chironomid data. Weighted averaging-partial least squares (WA-PLS; [62]) was performed to evaluate the idea of developing a chironomid-based training set applicable to the reconstructions of continentality. The continentality related variable which showed the strongest relationship to the chironomid assemblages and had a λ1:λ2 ratio of more than 1 in RDA was used. The strongest transfer function was determined as the one producing the lowest cross-validated root mean square error of prediction (RMSEP). The relevant components were accepted as statistically significant at the p ≤ 0.05 level. Bootstrapping techniques (9999 permutations; [60,63]) were used to estimate cross-validated error and performance statistics for the WA-PLS inference model, such as RMSEP, maximum and mean bias, and the coefficient of determination (R^2^) between inferred and predicted values.

Indicator species analysis (INDVAL; [64]) was applied to reveal characteristic morphotypes for the best performing continentality related variable according to the RDA results. Weighted-average regression with inverse deshrinking ([62]) was applied to the taxa that were determined as potential indicators to estimate taxon-specific continentality optima.

The software program R version 4.1.1. (R Core Team, 2021) was used to perform numerical analyses and create plots. The following packages were used: ‘tidyverse’ for data visualisation [65], ‘dplyr’ for data restructuring and basic calculations [66], ‘vegan’ for ordination and ANOSIM [67], ‘rioja’ for WA-PLS and WA and plotting the stratigraphic diagram [68], and ‘indicspecies’ for performing the INDVAL [69].

## Results

### Climatic and environmental setting of the dataset

**Fig 2:**
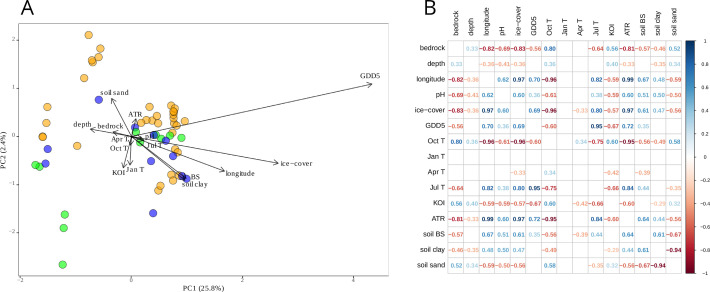
(A) Principal component analysis (PCA) with total variation of 27.6% and (B) Spearman correlation matrix of the climatic and environmental variables in the dataset: bedrock type; lake-water depth (m); longitude; lake-water pH; lake ice-cover (days); growing degree days with base temperature 5°C (GDD5); October (Oct T), January (Jan T), April (Apr T) and July (Jul T) mean air temperatures (°C); Kerner Oceanity Index (KOI); and annual temperature range (ATR); soil base saturation (soil BS); soil clay content (soil clay); soil sand content (soil sand). Continental sites (orange) are KOI −10–0, transitional (green) are KOI = 0–10, and oceanic (blue) are KOI 10–20.

PCA of the environmental and climate data indicated that GDD5 and ice-cover had the longest gradients (Fig 2A). Most tested variables, except for ATR, KOI, January and October mean temperatures, aligned with the first PCA axis, hence the explanatory power of the axis was high (25.8%). Among the climatic variables, GDD5, KOI, ATR, October and July air temperatures were significantly correlated with one another and with most of the remaining climatic variables. April air temperatures were correlated with only two climatic variables: KOI and ice-cover. January air temperatures explicit no significant correlations with other variables (Fig 2B). Environmental variables (water depth, pH, soil base saturation, sand and clay content) generally had correlation values of <± 0.7 with climatic variables (GDD5, ice-cover, July, April, and October mean air temperatures, ATR, KOI) except for bedrock which was highly correlated (>0.8) with ice-cover, ATR, and October mean air temperature.

### Chironomidae assemblage composition and distribution

**Fig 3:**
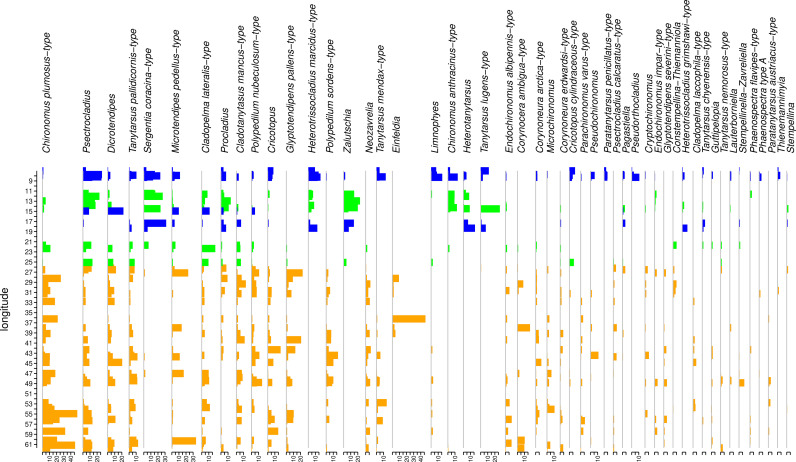
Chironomid morphotypes with abundances in the dataset of at least 2% in one sample. Species are arranged in abundance according to the longitudinal gradient. Continental sites (orange) are KOI −10–0, transitional (green) are KOI = 0–10, and oceanic (blue) are KOI 10–20.

The harmonized dataset includes on average 63 head capsules per sample with counts ranging from 42 head capsules to 139 head capsules. The dataset includes 51 lakes and 73 morphotypes (Fig 3; S1 File). The most abundant morphotypes are *Chironomus plumosus-*type (0.8–49.1% per sample), *Psectrocladius sordidellus-*type (0.7–26.9%), *Dicrotendipens* (0.7–22.2%), and *Tanytarsus pallidicornis-*type (0.7–12.8%). The presence of *Chironomus anthracinus*-type, *Heteratanytarsus*, *Heterotrissocladius marcidus*-type, *Sergentia coracina*-type, and *Zalutschia* characterises the transitional and oceanic parts of the dataset. These taxa are either absent or present in lower quantities in more continental lakes. Transitional lakes are further differentiated from oceanic ones by the presence of *Psecrocladius penicillatus*-type and *Pseudorthocladius*, and higher abundances of *Heterotrissocladius marcidus*-type, *Tanytarsus pallidicornis*-type and *Microtendipes pedellus*-type.

ANOSIM revealed that the chironomid assemblages can be significantly divided by KOI (p = 0.007) with an R-value of 0.24 indicative of some overlap in taxonomic composition between continental, transitional, and continental sites.

### Redundancy analysis

For the two tested continentality indices, KOI explained more variation in the chironomid assemblages than ATR (18.4% and 15% respectively; Table 2) with a λ_1_:λ_2_ ratio greater than 1. GDD5 and July mean air temperature explained the same amount of chironomid assemblage variation (17.2% and 17.4%, respectively; Table 2) and both have a λ_1_:λ_2_ ratio >1 and a stronger explanatory power compared to April (4%), January (8%) and October (14.2%) mean air temperatures. April and January mean air temperatures are the only variables aligned with the second RDA axis. The number of ice-cover days shows a significant influence on the chironomid assemblages (Table 2) with 15.5% of the variation explained and a λ_1_:λ_2_ ratio >1.

**Table 2:** Results of the redundancy analysis (RDA) of the dataset and tested environmental and climatic variables in the dataset: lake-water depth (m); longitude; lake-water pH; bedrock type; July (Jul T), October (Oct T), and April (Apr T) mean air temperatures (°C); lake ice-cover (days); growing degree days with base temperature of 5°C (GDD5); Kerner Oceanity Index (KOI); and annual temperature range (ATR). The proportion of chironomid-assemblage variation explained by each variable, *p*-values, and λ_1_:λ_2_ ratios are given.

Variable	% of variation explained	*p*-value	λ_1_:λ_2_
**Longitude**	13.1	0.001	0.8
**Environment**			
**Bedrock**	16.8	0.001	1.1
**pH**	12.3	0.001	1
**Soil base saturation**	8	0.002	0.5
**Soil clay content**	10	0.001	0.7
**Soil sand content**	4.7	0.013	0.2
**LOI (Russian and Latvian samples)**	–	0.6	–
**Depth (m)**	8.3	0.001	0.5
**Climate**			
**GDD5**	17.2	0.001	1.5
**Oct T**	14.2	0.001	1.2
**Apr T**	4	0.045	0.2
**Jul T**	17.4	0.001	1.3
**Jan T**	8	0.021	0.5
**Ice-cover**	15.5	0.001	1.1
**ATR**	15	0.001	1.3
**KOI**	18.4	0.001	1.6

**Fig 4:**
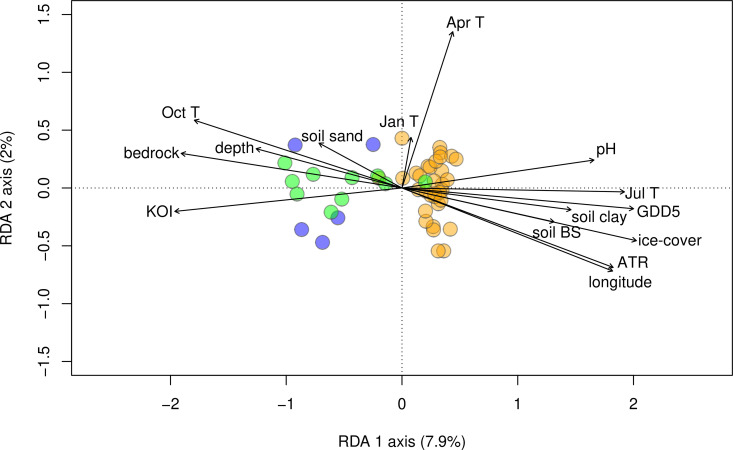
Redundancy analysis (RDA) plot showing the climate and environmental variables, revealed the significant dependency in the dataset: lake-water depth (m); longitude; lake water pH; bedrock type; soil base saturation (soil BS); soil clay content (soil clay); soil sand content (soil sand); July (Jul T), January (Jan T), October (Oct T), and April (Apr T) mean air temperatures (°C); lake-ice cover (days); growing degree days with base temperature of 5°C (GDD5); Kerner Oceanity Index (KOI); and annual temperature range (ATR). Variables explain 44.6% of variation in total with a *p*-value of 0.001. Continental sites (orange) correspond to −10–0 KOI, transitional sites (green) to 0–10 KOI, and oceanic sites (blue) to 10–20 KOI.

Bedrock explained 16.8% of the chironomid-assemblage variation (Table 2; Fig 4). Lake-water pH accounted for 12.3%, and lake-water depth explained 8.3% of the variation in the chironomid assemblages. LOI did not reveal any significant influence on chironomid assemblages (Russian and Latvian parts).

### Chironomid – Kerner Oceanity Index relationships

**Fig 5:**
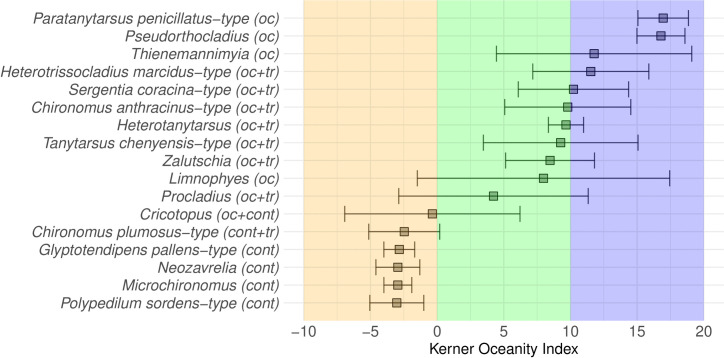
Weighted-average based Kerner Oceanity Index (KOI) optima and tolerances for morphotypes revealed as indicators by INDVAL. The continentality group affiliation identified by INDVAL is marked in brackets: oc – oceanic, tr – transitional, cont – continental; all taxa revealed statistical significance in the corresponding zone based on IndVal. The background is colored according to the KOI: continental (orange) for KOI < 0, transitional (green) for KOI 0–10, oceanic (blue) for KOI > 10.

INDVAL revealed significant indicator morphotypes for the continental, oceanic, and transitional (continental-transitional and oceanic-transitional) groups (Fig 5; S3 File). Continental sites are indicated by the presence of *Glyptotendipes pallens-*type, *Neozavrelia*, *Polypedilum sordens-*type, and *Microchironomus.* The continental-transitional morphotype is *Chironomus plumosus*-type. Oceanic sites are characterised by *Paratanytarsus penicillatus*-type, *Pseudorthocladius*, *Thienemannimyia*. Oceanic-transitional morphotypes are *Procladius*, *Heterotrissocladius marcidus*-type, *Sergentia coracina-*type, *Zalutschia*, *Chironomus anthracinus*-type, *Heterotanytarsus*, and *Tanytarsus chinyensis*-type. One morphotype (*Cricotopus)* is assigned to the continental-oceanic group.

Weighted-average regression (Fig 5; S4 File) reveals that the widest tolerance interval has morphotypes with optima in the transitional part of the dataset (*Procladius, Limnophyes, Tanytarsus chinyensis*-type) and *Thienemannimyia* from the oceanic part of the dataset. The smallest tolerances are shown by *Microchironomus* and *Glyptotendipes pallens*-type, both from the continental part of the dataset.

### Inference model for continentality reconstructions

**Fig 6:**
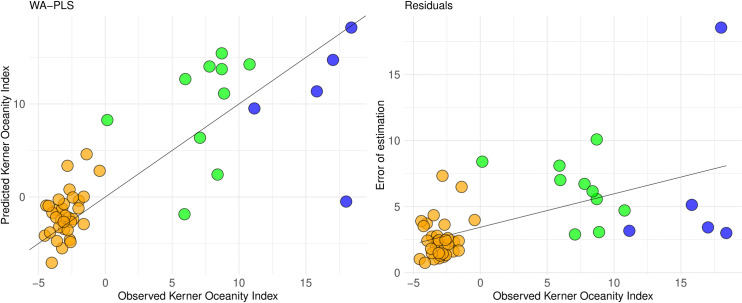
(A) Diagnostic plot of cross-validated estimates of the dataset compared with observed Kerner Oceanity Index (KOI) values and (B) residuals plot from a weighted-average partial least squares (WA-PLS) model based on two components. Continental sites (orange) correspond to −10–0 KOI, transitional sites (green) to 0–10 KOI, and oceanic sites (blue) to 10–20 KOI.

The KOI-based two-component WA-PLS inference model has an RMSEP of 5.1, RMSE of 4.3, R^2^ of 0.72, average bias of −0.1, and maximum bias of 14.6. A scatterplot of the cross-validated predicted vs. observed KOI generally follows a 1:1 relationship (Fig 6A). The reconstruction errors (Fig 6B) indicate an increased error in the transitional and oceanic parts of the dataset.

## Discussion

### Effect of environmental and climate variables on the chironomid assemblages

July mean air temperature, GDD5, ice-cover are positively correlated with each other (correlation index >0.8; Fig 2B) and are arranged together along the first axis of both the PCA and RDA plots (Fig 2A and 4). Also, their explanatory powers are relatively similar (Table 2). Thus, July mean air temperature, GDD5, ice-cover reflect the longitudinal gradient, and hence, their individual effects on chironomid assemblages are difficult to distinguish reliably. The discussed variables are positively correlated with the first axis. The positive correlation between bedrock and October mean air temperature is most probably an artefact of the dataset design. The climate variables that did not reveal strong (>0.7) correlations are lake-water pH, lake-water depth, April and January mean air temperature, and KOI. The independence of KOI can be explained by its formula: it includes October, January and April mean air temperatures. Further, KOI explains the highest amount of variation in the chironomid assemblages (18.4%; Table 2). Thus, KOI appears to be a key driver of change in the chironomid assemblages in the present dataset (Fig 4; Table 2). However, the chironomid assemblages from the dataset could be influenced by unmeasured environmental variables (e.g., water trophic state, conductivity, and oxygen content; catchment vegetation cover/type).

In our study, GDD5 has higher explanatory power (17.2%) than in previous studies: GDD5 explains 9% of the variation in Swiss Alps chironomid assemblages [70] and 9.7% in New Zealand Alps assemblages [71]. This could be due to the wide spatial spread of our sampling sites combined with the long GDD5 gradient in our dataset (Fig 2A) and suggests the importance of growing-season length for chironomid assemblages. The high performance of July, relatively high of October, and significant April mean air temperatures in the RDA, which are all related to growing season duration, also support this interpretation. Furthermore, April mean air temperature, while having the lowest explanatory power (4%) among the tested climatic variables, is aligned with the second RDA axis, together with January mean air temperatures suggesting the distinctive impact of the growing-season start on the chironomid assemblages. While, the relationship of chironomid assemblages and October air temperatures has not been studied before, its relatively high explanatory power suggests that the time of the autumn water column mixing may also influence chironomid assemblages. January mean air temperature has been found to impact chironomid assemblages and explained 5.2% in Swedish chironomid training set [45]. The higher influence of January mean air temperatures on the studied dataset (8%) is explained by a high range of this climate variable: from −14 °C in Urals to −1−0 °C in coastal Baltic area.

Ice cover is known to cause depletion in the dissolved oxygen content of lake water [16,72]. Thus, the importance of ice cover can be explained by affecting dissolved oxygen changes, to which chironomids are known to be sensitive [35,36,73,74]. Warmer air temperature combined with shorter ice-cover duration have been shown to increase lake water pH [75] over a longer period of time. Our dataset covers a long gradient of ice-cover duration (35−196 days) and ATR, which may explain why lake-water pH has a higher explanatory power in our dataset compared to other chironomid datasets [21,42]. The strong negative correlation between ice-cover and bedrock type (r = −0.83) suggests that variations in bedrock may also influence the water pH, in addition to the direct effects of ice-cover.

The high explanatory power of bedrock type (16.8% variation in chironomid assemblages) can be explained by the fact that bedrock influences water chemistry, soil, terrestrial vegetation type, productivity of the ecosystem, and catchment erosion processes [76–84]. The explanatory power of lake-water depth (8.3%) is considerably stronger than in other studies [21,42,85]. This may be due to the broad depth range in our dataset (1–45 m), whereas the datasets referenced above did not include lakes deeper than 21 m. Water depth influences chironomids via variations in water temperature, oxygen concentration, habitat structure, macrophytes presence and food quality and availability [37,86]. The properties of surrounding soils (base saturation, sand and clay content) most probably have an indirect effect on chironomid assemblages through changing the limnological conditions by regulating the drainage of organic and inorganic components and during catchment erosion processes [87–93]. The independence of lake-water depth and pH, as well as soil properties from climatic variables makes it possible to separate the effect of local environmental factors from regional climate factors on the chironomid assemblages.

### Morphotype-specific relationships with kerner oceanity index

Chironomid taxa identified as characteristic of the continental group (Fig. 5) are commonly identified as warm summer-related ones [21,39,42]. Our study indicates that a preference for warm summer temperatures is accompanied by a tolerance for a short growing season in these taxa. The only exception is *Neozavrelia*, which was previously considered as a cold stenothermic taxon [53]. However, according to recent findings from central, eastern, and northern Europe [21,42] it appears to be a warm-related one. The modern distribution of *Neozavrelia* taxon includes both extreme oceanic climates (Norway, Russian Far East, Japan) as well as highly continental ones (Eastern Siberia) [53,94]. *Glyptotendipes pallens*-type has been observed to tolerate severe winter conditions [95], which probably helps it to survive in continental climates. *Microchironomus*, *Polypedilum sordens*-type, and *Glyptotendipes* have been recorded emerging in March and April, even beneath the snow/ice or through ice cracks [96]. Such behaviour can be considered as an adaptation to cold winters [97]. Also, in continental conditions of rapid seasonal change and hot summers, the emergence in early spring helps to avoid extreme air heat during mating. *Chironomus plumosus*-type, identified as a continental to transitional morphotype, reveals another survival strategy: during winter diapause nearly all the larvae are in the fourth instar and start the active emergence in June in Russia [98]. Also, a study by Self et al. [24] found that *Chironomus plumosus*-type show significant responses to continentality. From a morphological perspective, a small body size (*Microchironomus, Naeozavrelia*) and pigmentation (*Glyptotendipes pallens-*type, *Chironomus plumosus*-type*, Polypedilum sordens*-type*, Neozavrelia*) can help to survive cold winter conditions [97].

Most of the oceanic and oceanic-transitional morphotypes reveal a cool summer-related distribution [21,39,42]. Preference of a longer growing season is suggested by our data. Self et al. [24] identified *Heterotrissocladius marcidus*-type, *Heterotanytarsus*, *Sergentia coracina*-type, *Pseudorthocladius*, and *Thienemannimyia* as low continentality (oceanity) dependent taxa, which aligns well with our results. Also, these authors explain the distribution of *Pseudorthocladius* and *Thienemannimyia* in oceanic sites with their terrestrial and splash zones habitats where melting, refreezing, or wind removal can expose the chironomids to cellular damage from repeated freeze-thaw cycles. Terrestrial chironomids have adapted to such disturbances through behavioral and physiological mechanisms, including hibernation and the ability to lower their body’s freezing point [97]. Terrestrial *Limnophyes*, which is oceanic climate related in our dataset, is expected to have the same adaptations. *Heterotrissocladius marcidus*-type [99], *Sergentia coracina*-type [100], *Chironomus anthracinus*-type, *Procladius* [95], and *Tanytarsus chinyensis*-type [101] emerge in mid-summer and in autumn (September-October), and are thus adapted to oceanic cool springs, mild summers, and warm autumns.

### Creating a kerner oceanity index-based model

Two continentality indices (ATR, KOI) were tested in this study. ATR is calculated as the difference between the coldest and the warmest months, while the KOI calculation uses the ATR value and incorporates spring (April) and autumn (October) temperatures. A previous chironomid-continentality study [24] used the Gorzynski continentality index, which is also ATR-based, but includes latitude. However, as our dataset is constrained to a narrow latitudinal band (Fig 1, Table 1), it is assumed that any impact of latitude would not be detectable. Furthermore, the Gorczynski continentality index is not applicable to oceanic sites [102] and cannot therefore be used for coastal areas of the eastern Baltic and southern Scandinavia.

The RMSEP of the chironomid-inferred WA-PLS KOI model is 5.1. Considering that the RMSEP covers about 15% of KOI gradient length in the dataset (−4.5–27.5; Table 1), this seems to be a very promising variable. However, no KOI-based model has been published so far. In Self et al. [24], where the Gorzynski continentality index was used, the reported R^2^ value was 0.73, which is the same as in our model. The prediction errors (Fig 6) tend to increase from the continental to oceanic part of the dataset; a pattern also seen in Self et al. [24]. The issue could be because our dataset is relatively small but covers a large geographical area, which is reflected in the species occurrence pattern, with only 66% of the species occurring in at least five sediment samples (10% of the investigated dataset). Also, the higher proportion of continental than transitional and oceanic sites, increases the robustness of the continental part of the model. An increase in the number of sites, especially transitional and oceanic ones, and in the density of the training set may improve the model’s accuracy.

July air temperature has previously been considered as the main driver to explain chironomid assemblage distribution [21,39,42,85]. The high amount of variation explained by July air temperature in our dataset, where the summer temperature gradient was intentionally reduced by the sampling design, highlights its importance for chironomid assemblages in the selected study area. However, the performance of the current dataset in the statistical tests indicates that chironomids may be used as a continentality proxy. Development of specialised training sets dedicated to specific parameters are an essential prerequisite for successful reconstructions of different palaeoenvironmental variables.

## Conclusions

Summer temperatures are commonly considered to be a key driver of chironomid assemblage patterns. However, in our dataset, collected along a transect from the oceanic Atlantic coast to the continental central Russia, July air temperature is the second strongest explanatory variable and a part of a general longitude-related group of variables, which also includes annual temperature range, ice-cover duration, and growing degree days (GDD5). The Kerner Oceanity Index (KOI) represents a distinct gradient in the dataset. It explains the highest variation in the chironomid assemblages and is independent of the other tested variables. Therefore, KOI is a comprehensive continentality metric in our dataset, accounting for both the annual temperature range and the spring and autumn air temperatures.

Despite the dataset being relatively small for creating a robust palaeoclimate continentality reconstruction, the WA-PLS model performance for KOI shows promising results with an R^2^ = 0.73 and RMSEP of 5.1. We conclude, therefore, that further investigation of chironomid-continentality relationships and the creation of a larger continentality-based chironomid training set are justified.

## Supporting information

S1 File Chironomidae assemblages composition of the continentality dataset. (CSV)

S2 File Environmental data. (CSV)

S3 File IndVal performance values for the taxa revealed significancy. (DOCX)

S4 File Weighted average regression based estimated optima for Chironomidae morphotypes revealed significancy in IndVal. (CSV)
