# Supplementary material for: Chironomid-climate continentality conundrum
Source: PLoS One. 2025 Aug 5;20(8):e0327780. doi: 10.1371/journal.pone.0327780 (PMC12324118; doi:10.1371/journal.pone.0327780)
Supplement: S3 File — (DOCX) [file pone.0327780.s003.docx]

| Continentality group | Morphotype | stat | p-value |
| --- | --- | --- | --- |
| continental | *Glyptotendipens pallens-type* | 0.824 | 0.0056 |
| continental | *Neozavrelia* | 0.812 | 0.005 |
| continental | *Polypedilum sordens-type* | 0.758 | 0.014 |
| continental | *Microchironomus* | 0.651 | 0.0442 |
| oceanic | *Paratanytarsus penicillatus-type* | 0.707 | 0.0011 |
| oceanic | *Pseudorthocladius* | 0.707 | 0.0011 |
| oceanic | *Thienemannimyia* | 0.689 | 0.0025 |
| oceanic | *Limnophyes* | 0.627 | 0.0232 |
| continental+transitional | *Chironomus plumosus-type* | 0.944 | 2.00E-04 |
| oceanic+transitional | *Procladius* | 0.923 | 0.0001 |
| oceanic+transitional | *Heterotrissocladius marcidus-type* | 0.856 | 0.0002 |
| oceanic+transitional | *Sergentia coracina-type* | 0.772 | 0.0022 |
| oceanic+transitional | *Zalutschia* | 0.683 | 0.004 |
| oceanic+transitional | *Chironomus anthracinus-type* | 0.681 | 0.0049 |
| oceanic+transitional | *Heterotanytarsus* | 0.632 | 0.013 |
| oceanic+transitional | *Tanytarsus chyenensis-type* | 0.569 | 0.0258 |

INDVAL output
